# Novel molecular, structural and evolutionary characteristics of the phosphoketolases from bifidobacteria and *Coriobacteriales*

**DOI:** 10.1371/journal.pone.0172176

**Published:** 2017-02-17

**Authors:** Radhey S. Gupta, Anish Nanda, Bijendra Khadka

**Affiliations:** Department of Biochemistry and Biomedical Sciences, McMaster University, Hamilton, Ontario, Canada; Russian Academy of Medical Sciences, RUSSIAN FEDERATION

## Abstract

Members from the order *Bifidobacteriales*, which include many species exhibiting health promoting effects, differ from all other organisms in using a unique pathway for carbohydrate metabolism, known as the “bifid shunt”, which utilizes the enzyme phosphoketolase (PK) to carry out the phosphorolysis of both fructose-6-phosphate (F6P) and xylulose-5-phosphate (X5P). In contrast to bifidobacteria, the PKs found in other organisms (referred to XPK) are able to metabolize primarily X5P and show very little activity towards F6P. Presently, very little is known about the molecular or biochemical basis of the differences in the two forms of PKs. Comparative analyses of PK sequences from different organisms reported here have identified multiple high-specific sequence features in the forms of conserved signature inserts and deletions (CSIs) in the PK sequences that clearly distinguish the X5P/F6P phosphoketolases (XFPK) of bifidobacteria from the XPK homologs found in most other organisms. Interestingly, most of the molecular signatures that are specific for the XFPK from bifidobacteria are also shared by the PK homologs from the *Coriobacteriales* order of Actinobacteria. Similarly to the *Bifidobacteriales*, the order *Coriobacteriales* is also made up of commensal organisms, that are saccharolytic and able to metabolize wide variety of carbohydrates, producing lactate and other metabolites. Phylogenetic studies provide evidence that the XFPK from bifidobacteria are specifically related to those found in the *Coriobacteriales* and suggest that the gene for PK (XFPK) was horizontally transferred between these two groups. A number of the identified CSIs in the XFPK sequence, which serve to distinguish the XFPK homologs from XPK homologs, are located at the subunit interface in the structure of the XFPK dimer protein. The results of protein modelling and subunit docking studies indicate that these CSIs are involved in the formation/stabilization of the protein dimer. The significance of these observations regarding the differences in the activities of the XFPK and XPK homologs are discussed. Additionally, this work also discusses the significance of the XFPK-like homologs, similar to those found in bifidobacteria, in the order *Coriobacteriales*.

## Introduction

Bifidobacteria are an important group of commensal microorganisms comprising significant constituents of the gastrointestinal tracts of humans, other mammals, as well as insects [1–4]. These bacteria are able to metabolize a wide variety of carbohydrates and glycans, and their genomes are particularly rich in carbohydrates-utilizing enzymes [5–10]. The saccharolytic ability of bifidobacteria plays a major role in their adaptation as important, and often dominant, inhabitants of the gut microbiota and possibly also for their health-promoting effects [1,5,6,9,11–13]. Bifidobacteria differ from other gut microbes in terms of their fermentation of carbohydrates. Specifically, these organisms lack the enzymes aldolase and glucose-6-phosphate (G6P) NADP^+^ oxidoreductase, and as such, they are unable to utilize the conventional glycolysis pathway for carbohydrate metabolism [6]. Instead, bifidobacteria possess a unique fermentation pathway known as the “bifid shunt” which relies on the enzyme phosphoketolase to metabolize Fructose-6-phosphate (F6P) [3,14–19].

Phosphoketolases (PKs) are members of the thiamine pyrophosphate (TPP)-dependent enzyme family that play a crucial role in carbohydrate metabolism in various microbes [17,20]. Based on their substrate specificities, these enzymes can be categorized into two major groups. The common form of PK present in most organisms shows a substrate specificity mainly for xylulose-5-phosphate (X5P) (XPK, EC 4.1.2.9), and plays a fundamental role in pentose catabolism in obligatory and heterofermentative lactic acid bacteria (LAB), as well as in certain species of cyanobacteria and fungi via the phosphoketolase pathway [17,20–22]. In contrast, the form of PKs found in bifidobacteria (XFPK, EC 4.1.2.22) are unique in exhibiting comparable affinities for both X5P and F6P [16,18,23,24]. Thus, XFPK, in addition to splitting F6P into erythrose-4-phosphate and acetyl phosphate, also catalyzes the phosphorolysis of X5P into acetyl phosphate and D-glyceraldehyde-3-phosphate. Due to its ability to metabolize F6P, XFPK in bifidobacteria serves to link the carbohydrate metabolism pathway to the phosphoketolase pathway common to lactic acid group of bacteria [17,23,25–27]. Since the activity of PKs towards F6P is primarily found in bifidobacteria, the presence of the XFPK form of the enzyme is often used as a taxonomic tool for the identification of bifidobacteria [7,8,19,28,29].

Despite the well-known differences in the biological activities of PKs between bifidobacteria and other bacteria, very little is known about the molecular or biochemical basis accounting for the differences in the two forms of PKs. The amino acid sequences of XPK and XFPK exhibit more than 40% identity over their entire length, indicating that the two forms of enzymes are homologous [17,20,26]. The crystal structures of XFPK from *Bifidobacterium breve* and *Bifidobacterium longum*, with some bound cofactors and intermediates, have been solved [16,20]. The enzyme in bifidobacteria is a dimer with the active site located at the interface formed between the two subunits [16,20]. However, no structural information is available for the XPK form of the phosphoketolases.

Our recent comparative analyses of protein sequences from bifidobacteria have identified numerous conserved signature indels (CSIs) in various proteins involved in different cellular functions that are distinctive characteristics of the bifidobacterial homologs [30].

The present work focuses on the sequence features of the phosphoketolases to identify any characteristics that could prove helpful in understanding the differences between the two forms of PKs found in different organisms. These studies have led to the identification of multiple highly specific molecular differences in the forms of CSIs that clearly distinguish the XFPK of bifidobacteria from the XPK homologs found in most other bacteria. Interestingly, most of the molecular signatures that are specific for the XFPK from bifidobacteria are also shared by the PKs from the *Coriobacteriales* order of bacteria, which is comprised of saccharolytic organisms also belong to the phylum Actinobacteria [31,32]. Phylogenetic studies provide evidence that the PKs in bifidobacteria are specifically related to those found in the *Coriobacteriales*, suggesting that the gene for PK (XFPK) was horizontally transferred between these two groups. The results of protein modelling and *in silico* docking studies presented here reveal that a number of the identified CSIs in the PK sequences which are distinguishing characteristics of the *Bifidobacteriales/Coriobacteriales* homologs are present at the subunit interface in the XFPK structure and they are involved in the formation/stabilization of the protein dimer. The significance of these observations regarding the differences in the activities of the XFPK and XPK are discussed.

## Methods

### Identification of conserved indels and phylogenetic tree construction

Conserved signature indels (i.e. insertions or deletions) in the sequence alignment of phosphoketolase were identified as described recently [30,33]. A multiple sequence alignment of the PK homologs from representative bifidobacteria and other bacterial phyla was created using ClustalX [34]. The alignment was visually inspected for the presence of different indels that were flanked on both sides by at least 5–6 conserved amino acid residues in the neighbouring 30–40 amino acids. For all indels meeting these criteria, detailed BLASTp searches were carried out on short sequence segments containing the indel and the flanking conserved regions (60–100 amino acids long) to determine the specificity of the indels. SIG_CREATE and SIG_STYLE (available on Gleans.net) were used to create signature files that are shown here [30,33]. Due to space limitations, sequence information for all *Bifidobacterium* or *Coriobacteriales* species (or subspecies) is not shown in the alignment files. However, unless otherwise noted, all of the described CSIs are specific for the indicated groups (i.e. similar CSIs were not present in the protein homologs from other bacteria in the top 500 Blast hits) [30]. For phylogenetic analysis, a multiple sequence alignment of PK homologs was constructed from bifidobacteria, *Coriobacteriales* and a limited number of outgroup species (Firmicutes and Actinobacteria). After removing areas of poor sequence conservation using the Gblocks 0.91b program [35], a maximum likelihood (ML) tree based on the resulting alignment was constructed using the MEGA 6 program [36] employing the Jones-Taylor-Thornton [37] and Whelan and Goldman [38] substitution models, respectively.

### Structural analysis of the CSIs and homology modeling of phosphoketolase homologs

The structural models of the PK (or XFPK) protein from several bifidobacteria species (viz. *B*. *breve*, *B*. *bifidum*, *B*. *animalis*, *B*. *reuteri*) were generated using homology modelling. The secondary structure analyses on the selected homolog sequences were initially performed via PSIPRED v3.3 web server [39].The crystalized *B*. *longum* PK structure (PDB ID: 3AI7) was utilized as a template and the comparative modeling was carried out using MODELLER v9.11 [40]. Initially, 200 models were generated and ranked/selected using Discrete Optimized Protein Energy (DOPE) scores [41]. The secondary structure elements in the regions containing CSIs were examined and compared with results of the PSIPRED analysis to ensure their reliability. The stereo-chemical properties of the final models were assessed using three independent servers: RAMPAGE, ERRAT, and Verify3D [42], [43] [44,45]. These tools use a dataset of highly refined structures to evaluate the statistical significance of models based on the conformation, location, and the environment of each amino acid in the sequence, as well as the model’s overall structural stability. The superimposition of the validated models with the template structures was carried out using PyMOL (Version 1.7.4; Schrödinger, LLC.) to examine the structure and location of identified CSIs in the PK (or XFPK) structures..

Identification of the macromolecular interface formed between the individual subunit and the residues in the CSIs that are involved in subunit-subunit interactions was determined by submitting the three-dimensional coordinate file of the *B*. *longum* PFK dimeric structure to the PDBePISA server using default parameters (Version 1.48)[46].

### Protein-protein docking to examine the dimerization potential of the bifidobacteria PKs

The protein-protein docking approach was utilized to gain insights concerning the structural roles of the interface interacting residues and to study the dimerization potentials of the PK homologs from *Bifidobacterium* species. The structural models of the CSI-lacking forms of PKs from a number of bifidobacterial species (viz. *B*. *longum*, *B*. *breve*, *B*. *bifidum*, *B*. *animalis* and *B*. *reuteri*) were generated using homology modelling methods as described above. The known structures and the individual structural models of PFK monomers forms were submitted to three fully automated web-based protein-protein docking programs, viz. ZDOCK (Version 3.0.2)[47], PatchDock (Version Beta 1.3) [48], and ClusPro 2.0 [49] using default parameters. ZDOCK utilizes grid-based fast Fourier transform (FTT) for efficient global search of docking orientation between two proteins [47]. Its scoring function is based on pairwise shape complementarity, electrostatics, and a pairwise atomic statistical potential developed using contact propensities of transient protein complexes. PatchDock is a very efficient geometry-based molecular docking algorithm which is aimed to yield the good molecular shape complementarity of protein-protein complexes [48]. Its scoring function includes both geometric fit and atomic desolvation energy [48]. ClusPro utilizes PIPER, a rigid body docking program [50], which is based on a novel FFT based docking approach with pairwise potential [49,50]. The structures with maximum cluster size and the conformation closest to the solved crystal structure of PFK dimer with lowest root mean square deviation (RMSD) was selected as a representative structure for the detailed interface interaction analysis. Visualization and structure alignment of the CSI-containing and CSI-lacking dimer structure was carried out using PyMOL (Version 1.7.4; Schrödinger, LLC.). PDBePISA (Version 1.48)[46] server was used for detailed interface analysis.

## Results

### Distinguishing features of the phosphoketolase sequences from *Bifidobacteriales* and *Coriobacteriales*

**Fig 1:**
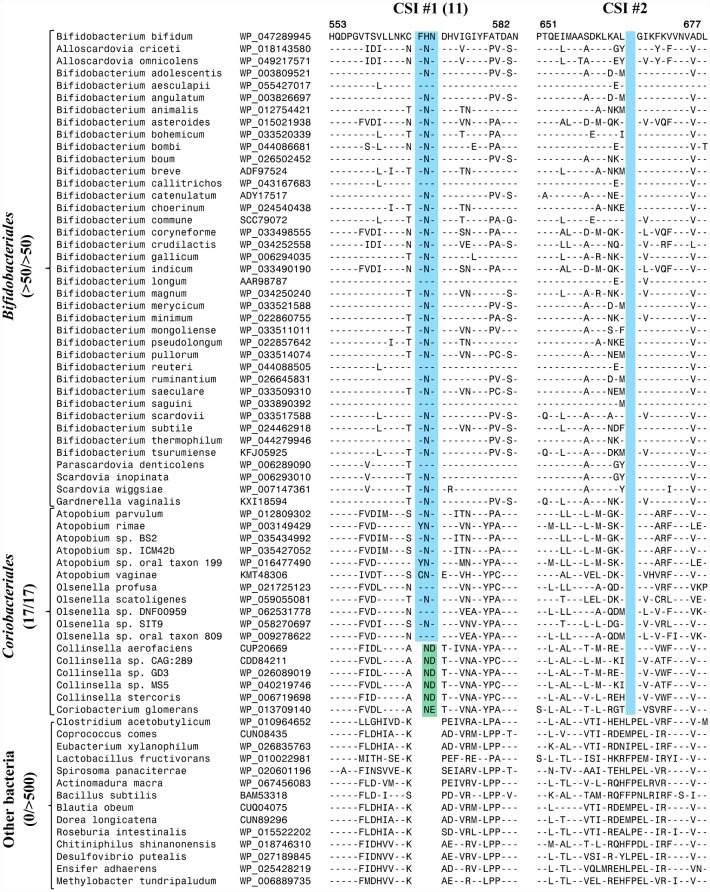
Excerpts from a sequence alignment of phosphoketolases showing a number of conserved signature indels (CSIs) that are either uniquely found in members of the orders *Bifidobacteriales* and *Coriobacteriales* or are commonly shared by the members of these two orders. For the CSI # 1(11) shown in this figure, CSI #1 refers to the 3 aa insert that is specific for the bifidobacteria and most *Coriobacteriales*, whereas the CSI # 11 corresponds to the 2 aa insert present in the same position in members of the genera *Collinsellla* and *Coriobacterium*. The dashes (-) in this alignment as well as in all other alignment figures indicate identity with the amino acid on the top line. Sequence information is presented for only a limited number of species. However, unless otherwise indicated the described CSIs are specific for the indicated groups of bacteria.

The PKs from bifidobacteria differ from other studied bacteria because of their ability to utilize/metabolize both F6P and X5P. To gain insights into the molecular basis of the differences in the biochemical properties of PKs from bifidobacteria (XFPK) versus other bacteria (XPK), a multiple sequence alignment of representative PK homologs from different bacterial groups was constructed. Examination of this sequence alignment has identified a number of conserved indels that are uniquely present in the PK homologs from bifidobacteria as well as those from members of the order *Coriobacteriales*, but not found in the homologs from other groups/phyla of bacteria. In Fig 1, we present excerpts from sequence alignment of PKs showing a number of conserved signatures indels (CSIs) found in this protein that are distinctive characteristics of the *Bifiodobacteriales/Coriobacteriales* homologs. The first of these CSIs (CSI #1(11)) is a 3 aa insertion that is commonly shared by all *Bifidiobacteriales* homologs (all available homologs without any exception) as well as different *Coriobacteriales* homologs. Within the *Coriobacteriales*, while the *Atopobium* and *Olsenella* spp. contain a 3 aa insertion similar to that found in the bifidobacteria, a shorter 2 aa insert is present in the *Collinsella* and *Coriobacterium* spp. (the shorter insert found in the latter taxa at the same position is referred to as CSI #11). The second CSI shown on the right hand side in Fig 1 (CSI # 2) is comprised of a 2 aa deletion that is specifically found in all *Bifidobacteriales* and *Coriobacteriales* homologs. Both of the identified CSIs are flanked by conserved regions and, except for their shared presence in all sequenced *Bifidobacteriales/Coriobacteriales* homologs, they are not found in homologs from any other bacteria (within the top 500 Blast hits). In addition to the CSIs shown in Fig 1, 4 other CSIs were identified in the sequence alignments of PKs (CSIs # 3–6), which are also commonly shared by all PK homologs from the *Bifidobacteriales/Coriobacteriales*. Sequence information for these CSIs (#3–6) is provided in Figures A, B and C in S1 Fig. These CSIs are also either uniquely or mainly found in the PK homologs from *Bifidobacteriales* and *Coriobacteriales*. However, in some of these cases, CSIs of similar lengths are also present in a limited number (≈ 5%) of other unrelated bacteria.

**Fig 2:**
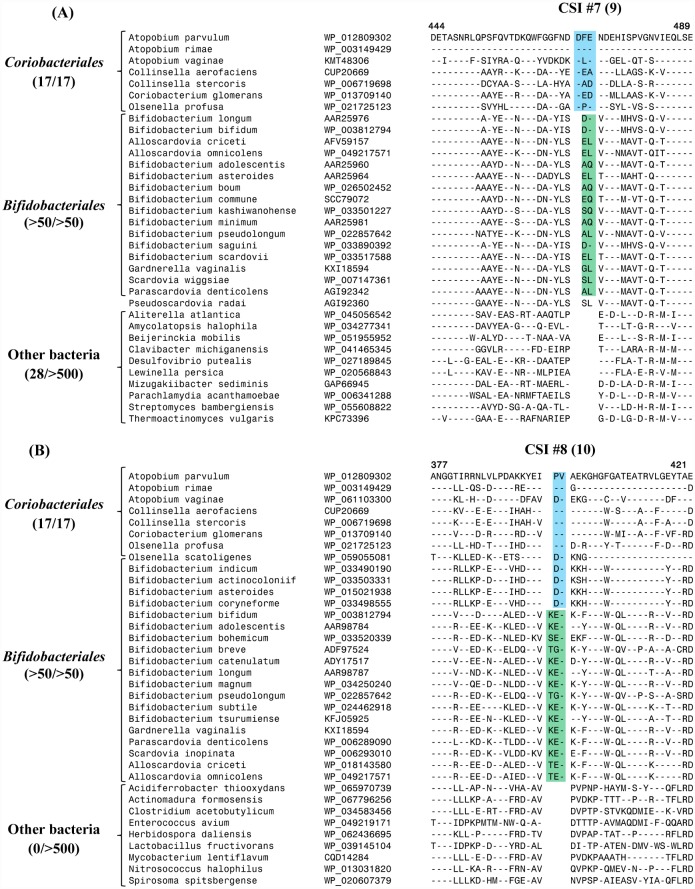
Partial sequence alignments of phosphoketolases showing a number of conserved signature indels (CSIs) where indels of different lengths are present in the same positions in members of the orders *Bifidobacteriales* and *Coriobacteriales*. When two different CSIs are present in the same position, in our numbering scheme, the first number refers to the CSI found in *Bifidobacteriales* group, whereas the second number in parenthesis describes the CSI found in the *Coriobacteriales*. Thus, CSI #7 and CSI #8 shown in this figure (parts A and B) refer to the indels present in all or most *Bifidobacteriales*, whereas CSI #9 and CSI #10 describe the indels found either only in the *Coriobacteriales* or in the *Coriobacteriales* plus certain deep branching bifidobacteria. The dashes (-) in the alignment indicate identity with the amino acid on the top line. The evolutionary interpretation of these indels is provided in the text and in Fig 3.

The sequence alignment of PK sequences also contains a number of additional CSIs where inserts of different lengths are present in the same positions in the *Bifidobacteriales* and *Coriobacteriales* homologs. These CSIs permit differentiation among the PK homologs found in these two orders of bacteria, and also between certain members of these two orders. In Fig 2A, sequence information is presented for a conserved region where a 2 aa insert is present in all of the PK homologs from bifidobacteria (CSI #7), whereas the homologs from *Coriobacteriales* contain a 3 aa long insertion (CSI #9) in the same position. In another location within the sequence alignment of PKs (Fig 2B), a 3 aa insert is present in the PK homologs of most bifidobacteria (CSI #8), whereas the homologs from *Coriobacteriales*, as well as a number of deep-branching members of the order *Bifidobacteriales*, were found to contain a 2 aa insertion in the same position (CSI #10). Lastly, one additional large CSI (11 aa long insertion) present in the PK homologs is only found in the *Coribacteriales* homologs belonging to the genera *Collinsella*, *Coriobacterium* and *Olsenella*, but it is not found in members of the genus *Atopobium* or *Bifidobacteriales* species. Sequence information for this CSI (CSI #12), is presented in Figure D in S1 Fig.

### Phylogenetic branching pattern of the PKs indicate horizontal gene transfer from *Coriobacteriales* to the *Bifidobacteriales*

**Fig 3:**
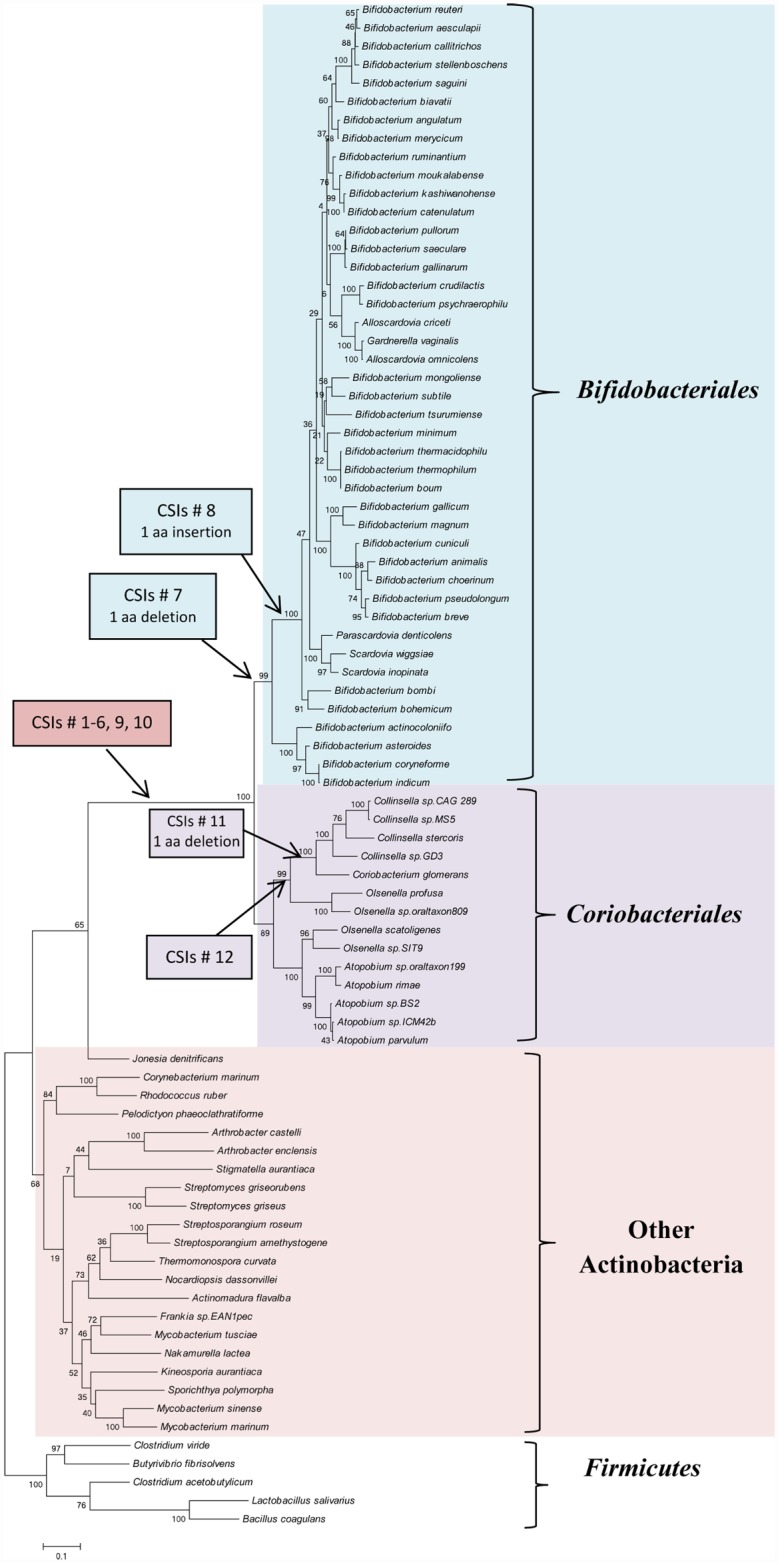
A maximum likelihood distance tree based on PKs sequences for members of the phylum Actinobacteria are representative outgroup species from the phylum *Firmicutes*. The numbers on the nodes indicate bootstrap scores for the group of species represented by different nodes. In this tree, members of the order *Bifidobacteriales* branch with the *Coriobacteriales* and the clade comprising of these two orders is separated from all other Actinobacteria/bacteria by a long branch. Based on the species distributions of different CSIs, the evolutionary stages where genetic changes giving rise to different CSIs have likely occurred are marked.

The shared presence of multiple CSIs by the PFK homologs from bifidobacteria and *Coriobacteriales* strongly suggests that the homologs from these two groups are closely related. Although the orders *Bifidobacteriales* and *Coriobacteriales* are both part of the phylum Actinobacteria, in phylogenetic trees based on 16S rRNA and other genes/proteins sequences, members of these two orders exhibit distinct branching [51–54]. In contrast to the *Bifidobacteriales*, which branch in the proximity of the order *Actinomycetales*, the *Coriobacteriales* species, along with the other members of the class *Coriobacteriia*, form one of the deepest branching lineages within Actinobacteria [51,53,54]. To understand the significance of the shared presence of multiple highly-specific sequence features by these two groups of bacteria, a phylogenetic tree based on the sequences of PK homologs was constructed. The maximum-likelihood tree based on PK sequences, shown in Fig 3, contains information for all bifidobacteria and *Coriobacteriales* homologs as well as limited representatives from other orders of Actinobacteria, and also some sequences from the deeper branching Firmicutes phylum. In this tree, which was rooted using sequences from the Firmicutes species, the homologs from bifidobacteria and *Coriobacteriales* formed a strongly supported clade, which branched deeply in comparison to the homologs from other actinobacteria, and this clade was separated from all other bacteria by a long branch. The observed branching and the strong affinity of the bifidobacterial homologs with the *Coriobacteriales* in the PK tree is in contrast to the distinct branching of the members of these two orders in the 16S rRNA tree and phylogenetic trees based on other genes/proteins sequences [51,53,54]. The observed results strongly suggests that the gene for the PK has been horizontally transferred between these two orders of Actinobacteria, and based on the known deeper branching of the order *Coriobacteriales* [31,51,53], the gene transfer has likely occurred from a *Coriobacteriales* to the *Bifidobacteriales*.

The inference from phylogenetic studies that the PK gene in *Bifidobacteriales* has been acquired from *Coriobacteriales* permits us to offer the most parsimonious explanation for the species distribution of different CSIs that are found in the PK homologs from these two groups of bacteria. Thus, the CSIs #1–6, where CSIs of similar lengths are present in different *Coriobacteriales* and *Bifidobacteriales* homologs, were likely present in the transferred *Coriobacteriales* PK gene. It can also now be inferred that the transferred *Coriobacteriales* PK gene contained a 3 aa insertion where the CSI #7(9) is found and an insertion of 2 aa, where the CSI #8(10) is present (Fig 2). Subsequent to the acquisition of this PK gene by a common ancestor of the *Bifidobacteriales*, further changes have occurred in this region that account for the differences in the lengths of the CSI #7 versus CSI #9, and CSI #8 versus CSI #10 between the *Coriobacteriales* and the *Bifidobacteriales*. These changes include a 1 aa deletion in the PK gene in the common ancestor of bifidobacteria where the CSI #7 is found, and a 1 aa insertion in the PK gene in the common ancestor of bifidobacteria, except the deepest branching members, where the CSI # 8 is found. The species specificities of different identified CSIs and the evolutionary stages where the genetic changes which gave rise to these CSIs have likely occurred are marked in the phylogenetic tree shown in Fig 3.

The species distributions of different CSIs also provide insights concerning the *Coriobacteriales* taxa from which the PK gene was likely transferred to the *Bifidobacteriales*. Of the described CSIs, the CSI #12 (Figure D in S1 Fig) is a specific characteristic of the PK homologs belonging to the genera *Collinsella*, *Coriobacterium* and *Olsenella*, but it is lacking in members of the genus *Atopobium*. The absence of this large CSI in all *Bifidobacteriales* homologs provides evidence that the PK gene was not acquired from members of the order *Coriobacteriales* which contain this CSI, but instead it originated from a member of this order lacking this CSI, such as a members of the genus *Atopobium* or closely related taxa. The CSI #1(11) (Fig 1) where a 3 aa insertion is found in all *Coriobacteriales* and *Bifidobacteriales* PK homologs, except those from the genera *Collinsella* and *Coriobacterium* also provides evidence that the transferred PK gene was not derived from these two genera of the *Coriobacteriales*.

### Locations of the CSIs in the phosphoketolase structure and their possible significance

**Fig 4:**
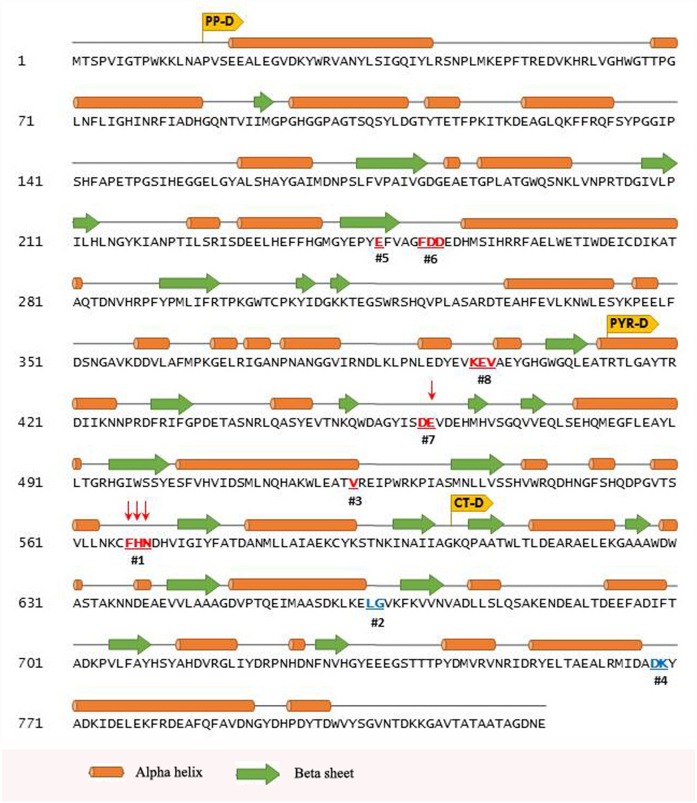
Primary sequence of phosphoketolase protein from *Bifidobacterium longum* depicting the location of different CSIs. Secondary structure elements are displayed above the primary sequence, with helices shown as cylinders and sheets shown as arrows. The secondary structure information was obtained directly from the solved structure of *B*. *longum* PK from Protein Data Bank (PDB ID: 3AI7). NCBI CD-search webserver was used to demarcate the three domains (yellow boxes): N-terminal PP-domain (PP-D), middle PYR-domain (PYR-D), and the C-terminal domain (CT-D). The eight identified CSIs in bifidobacteria PKs are indicated by bold and underlined residues, insertions are shown in red and the positions where deletions are present are shown in blue. The arrows on the top of CSIs indicate the residues contributing in subunit-subunit interactions, as determined via the PISA webserver.

The phosphoketolase protein is comprised of three domains: N-terminal PP-domain (PP-D), middle PYR-domain (PYR-D), and the C-terminal domain (CT-D). The locations of the different identified CSIs in the primary structure of the *B*. *longum* protein are depicted in Fig 4. The insertions in the PK sequence in this figure are indicated by red-colored bold and underlined residues, whereas the deletions are present in between the residues marked in blue. Secondary structure elements of the sequence are displayed above the primary sequence, with helices shown as cylinders and sheets shown as arrows. As seen, the identified CSIs are present in different domains of the bifidobacteria PK homologs.

**Fig 5:**
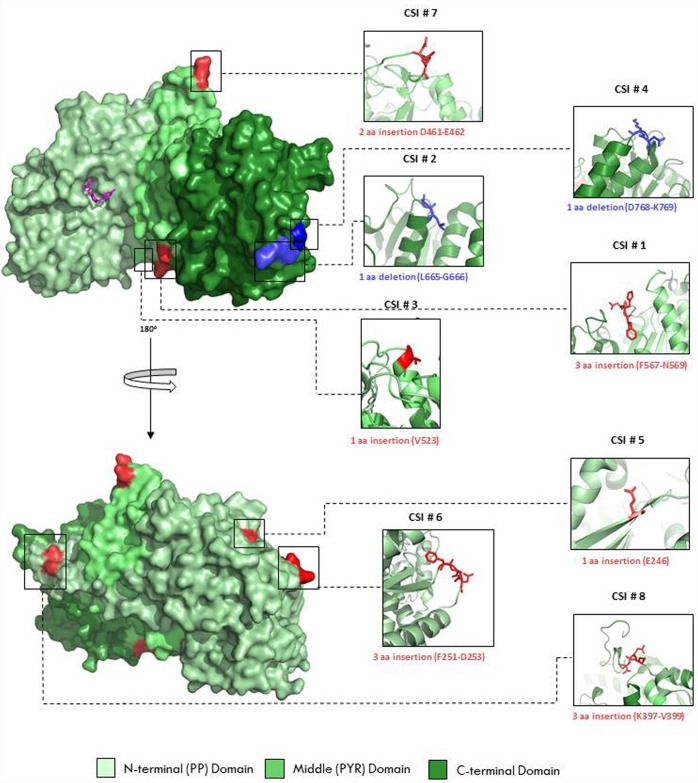
Surface representation of the phosphoketolase crystal structure monomer from *Bifidobacterium longum* (PDB ID: 3AI7). The three different domains in the protein are shown in three different shades of green color; pale green as N-terminal (PP) domain, lime green as Middle (PYR) domain, and forest green as C-terminal domain. The bound TPP cofactor located at the active site is shown in magenta. Close-up views of the regions containing eight different Indels are shown in cartoon representation with the insertions are depicted as red and the deletions as blue. The locations of the two deletions are highlighted by coloring the flanking residues as blue.

We have also mapped the locations of different CSIs in the crystal structure of PK from *B*. *longum* and a surface representation of the CSIs in the structure of a PK monomer (PDB ID: 3AI7) is shown in Fig 5. The three different domains in the protein are shown in three different shades of green color; pale green as the N-terminal (PP) domain, lime green as the middle (PYR) domain, and forest green as the C-terminal domain. The bound TPP cofactor located at the active site is shown in magenta. Close-up views of the regions of the PK protein containing the eight bifidobacteria CSIs are shown in cartoon representation with the insertions depicted in red and the deletions in blue (Fig 5). As seen from Figs 4 and 5, most of the identified CSIs in the PK protein are present in between the secondary structure elements found in the protein and most of them are located on the surface of the PK monomer. The only exceptions seen are CSIs #3 and #5, where single amino acid insertions have occurred at the end of a helix or beta sheet leading to possible lengthening of these structural elements.

**Fig 6:**
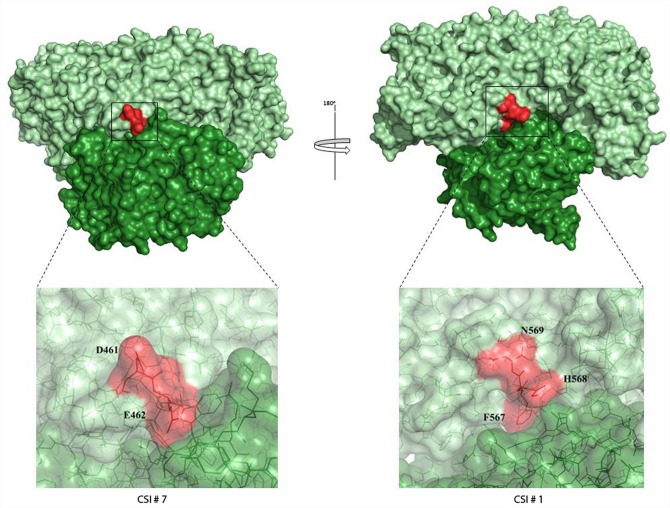
Surface representation of the phosphoketolase crystal structure dimer from *Bifidobacterium longum* (PDB ID: 3AI7). Individual monomers are shown in two different shades of green (pale green and forest green). The residues from two different CSIs (#1 and # 7) that are indicated to be involved in the dimer formation are highlighted red. The lower figures show close-up views of the 2 aa (CSI # 7) (left; D461-E462) and the 3aa insertion (CSI # 1) (right, F567-N569) (shown in red). Individual residues are labelled and clearly show their close proximity to the other subunit.

The functional PK enzyme in bifidobacteria is a dimer with the active site located between the subunit interface. To explore the macromolecular interface formed between the individual monomers and to determine if any of the CSIs in the bifidobacteria PK are involved in the interaction between the subunits, the structural coordinate file for one of the PK subunit from *B*. *longum* (PDB ID: 3AI7) was submitted to the PDBePISA server (Version 1.48; Krissinel and Henrick, 2007). A surface representation of the phosphoketolase dimer from *B*. *longum* showing the subunit interaction is shown in Fig 6. Individual monomers in this figure are shown in two different shades of green (pale green and forest green). As seen from Fig 6A, the residues from two different CSIs (shown in red) are located at the subunit interface and are indicated to be involved in dimer formation. Close-up views of the two CSIs which are located at the subunit interface, viz. CSI #7 (D461-E462) and CSI #1 (F567-N569), are shown in Fig 6B. Of the CSIs located at the interface, several residues are involved in specific interactions; GLU (E) at position 462 is involved in hydrogen bonding, PHE (F) at position 567 is an interface residue, and HIS (H) at position 568 is involved in salt bridge formation.

**Fig 7:**
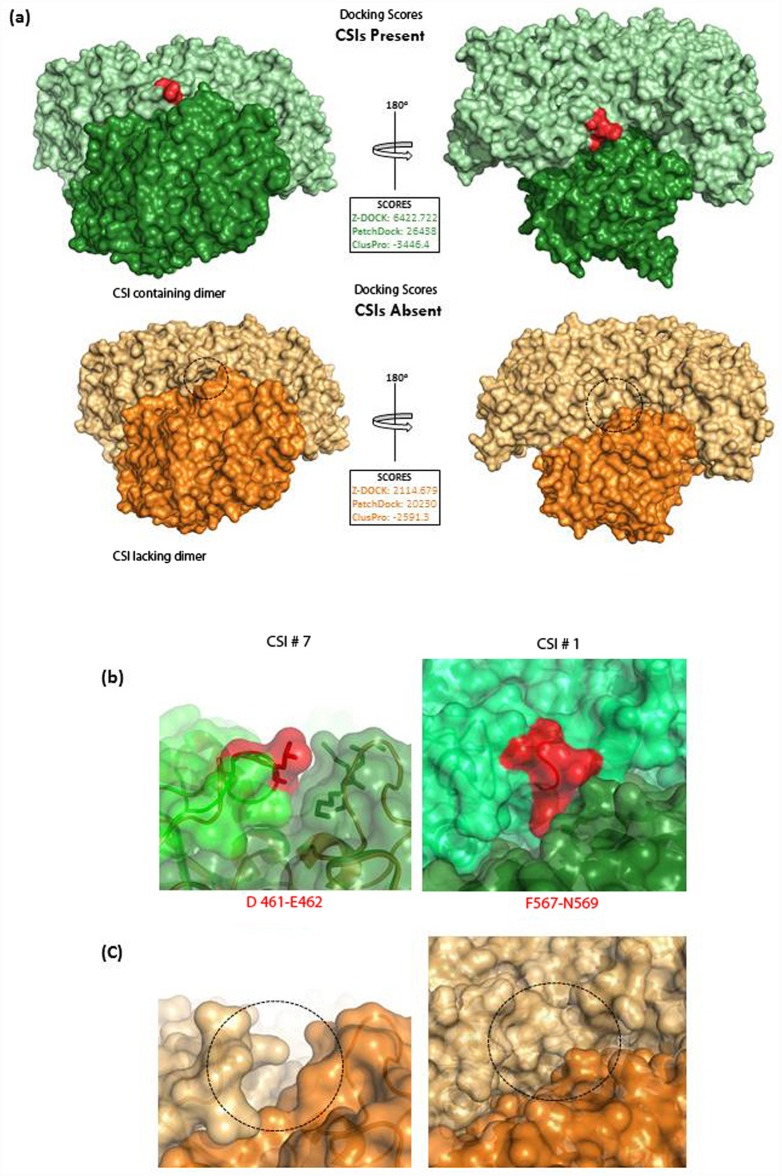
**(a) The crystal structure of *Bifidobacterium breve* phosphoketolase dimer (PDB ID: 3AHC; green) with all CSIs present in the structure (red) versus model structure of the *B*. *breve* phosphoketolase dimer with the CSIs #1 and # 7 removed (orange).** The dimers were generated by submitting the individual monomers (crystal structures and homology models) to three webservers: Z-DOCK, PatchDock, and ClusPro. The difference in docking scores generated by these servers is shown. The representative dimer PK from *B*. *berve* was calculated using ZDOCk. Removal of the CSIs resulted in small cavities along the dimer interface (indicated by dashed circle) and a significantly reduced score. **(b)** Close-up views of the residues from 2 aa insertion and 3 aa insertion show that these CSIs are located at the dimer interface and directly involved in interactions with the other subunit (indicated by an arrow). **(c)** Close-up views of the region in the protein from where the residues corresponding to 2 aa insertion (CSI # 7) and 3 aa insertion (CSI # 1) were removed. The gaps created by the removal of these CSI are indicated by dashed circle.

To explore the roles of the CSIs #1 and #7 in the formation/stabilization of the PK dimers in bifidobacteria, dimerization potentials of the bifidobacterial PK homologs with and without these CSIs were investigated by means of protein-protein docking studies. For these studies, both the known structures, as well as the validated homology models of PK from several bifidobacteria species which either contained or lacked the CSIs # 1 and #7, were submitted to the three online protein docking servers: ZDOCK (Version 3.0.2; Pierce et al., 2011), PatchDock (Version Beta 1.3; Duhovny et al., 2002), and ClusPro 2.0 (Comeau et al., 2004). The docking scores obtained from the three different servers are shown in the Table 1. As seen from Table 1, the docking scores (i.e. dimerization potentials) of PFK homologs that contained the CSIs were much higher in comparison to those obtained with the CSI-lacking homologs, and all three docking servers yielded similar results. The results from the ZDOCK-server, which consistently produced dimer conformations with lower RMSD values compared to the other servers, were then uploaded to PDBePISA (Version 1.48; Krissinel and Henrick, 2007) for detailed interface analysis. The representative structure of *B*. *breve* PFK dimer structure containing CSIs as well as lacking the CSI #1 and CSI #7, obtained from ZDOCK is shown in the Fig 7. As shown in Fig 7, the residues in the CSI #1 and CSI #7 (labelled and highlighted red) provide additional surface area for binding and interaction at the interface and for dimer formation. The removal of the residues corresponding to these CSIs in the region resulted in the loss of an interacting surface at the interface (Fig 7c).

**Table 1:** Protein-protein docking results of *Bifidobacterium* XFPK structure models for the CSIs-containing and CSIs-lacking proteins.

	CSI-containing Structure			CSI-lacking Structure		
Homolog	ZD	PD	CP	ZD	PD	CP
***Bifidobacterium bifidum***	5,565.215	39,902	-3,141.1	1,331.985	39,648	-2,301.8
***Bifidobacterium animalis***	3,206.406	54,118	-2,586.3	2,195.55	25,188	-,2,355
***Bifidobacterium longum***	3,217.031	48,084	-3,673.6	2,044.969	28,778	-2,572.4
***Bifidobacterium reuteri***	4947.682	51,414	-2,820.4	2,974.984	45,922	-2,609.8
***Bifidobacterium breve***	6,422.722	26, 438	-3,446.4	2,114.679	20,230	-2,591.3

Three different servers viz. Z-DOCK score (ZD), PatchDock score (PD), and ClusPro (CP) were utilized to create the dimer complex of PFK. The docking results are shown as Z-DOCK score, PatchDock geometry shape complementary score, and ClusPro (CP) lowest energy score (negative value). The removal of the CSIs # 1 and #7 from PFK homolog structures from *Bifidobacterium* species resulted in a dimer with decreased docking score when compared to the docking scores of unmodified (CSI-containing) proteins, as determined by all three servers.

## Discussion

Bifidobacteria differ from all other microbes in using a unique fermentation pathway known as the “bifid shunt” for the metabolism of different carbohydrates [5–7,14]. A key component of the “bifid shunt” enabling carbohydrate metabolism via this pathway is the presence of a novel form of the enzyme phosphoketolase (XFPK), which, in addition to carrying out phosphorolysis of X5P, is also able to convert F6P into erythrose-4-phosphate and acetyl phosphate [18,20,24,29]. The existence of the bifid shunt allows bifidobacteria to produce more ATP from carbohydrates than through other conventional pathways [5,6,20]. Specifically, the bifid shunt yields 2.5 ATP per mole of glucose compared to 2 ATP per glucose formed via the Embden-Meyerhof-Parnas glycolytic pathway [5,6,20]. Each mole of glucose also leads to the formation of 1.5 moles of acetate and 1 mole of lactate. The formation of these metabolites is of great benefit to the host organisms; the acetate produced in the gut is transported to the liver and used for the production of ATP, whereas lactate possesses anti-microbial activity and prevents proliferation of potential pathogens [5,5,6,20,55,56].

Phosphoketolases exhibiting high degree of sequence similarity to the bifidobacterial XFPK are widely distributed among prokaryotic organisms, and certain eukaryotes, but they exhibit specificity for only X5P and are unable to metabolize F6P [17,22,26,57–59]. However, the molecular and/or structural characteristics that differentiate the XFPK from XPK, which may be responsible for the important differences in their biochemical properties, are not known at present. Analyses of PK sequences from different organisms carried out in this work have provided important insights in this regard. Based on comparative analyses of PK sequences, this work has identified multiple high-specific sequence features in the forms of CSIs in the PK sequences that clearly distinguish the XFPK homologs of bifidobacteria from the XPK homologs found in most other organisms. An interesting and unexpected result is the discovery that the XFPK homologs from bifidobacteria are closely related to those found in the order *Coriobacteriales* and that most of the CSIs that are distinctive characteristics of the bifidobacteria XFPK are also present in the *Coriobacteriales* PKs. Phylogenetic studies on PK sequences show that the homologs from bifidobacteria from a strongly supported clade with the *Coribacteriales* PKs and the observed branching pattern of species from these two orders is different than that seen in phylogenetic trees based on other gene/protein sequences [51,53,54]. The observed branching pattern strongly suggests a horizontal transfer of the PK gene between these two orders of Actinobacteria. The phylogenetic branching pattern and the species distribution of different identified CSIs suggest that the PK gene was horizontally transferred from a *Coriobacteriales* to the common ancestor of the *Bifidobacteriales* that the *Coriobacteriales* taxon from which the PK gene was acquired likely corresponded to a member of the genus *Atopobium* or a closely related species.

The findings from this study indicate that XFPK homologs from bifidobacteria differ from all other XPK homologs (except those from the *Coriobacteriales*) by many highly-conserved sequence features and they strongly suggests that the described sequence characteristics should play an important role in the observed differences in the biochemical characteristics of the XFPK and XPK homologs. The identified conserved indels are present in different regions of the XFPK protein sequence, and their structural analysis reveals that all of the identified CSIs, except possibly two, are located in the surface loops of XFPK. Earlier work on conserved indels provides evidence that the genetic changes represented by such indels are essential for the proper functioning of the proteins in the CSI-containing organisms, and the removal of such CSIs has detrimental effect on the proper functioning of the concerned proteins [60]. The localization of CSIs within surface loops of the proteins has also been noted in a number of previous studies [30,61,62]. The surface loops in protein sequences constitute highly accessible regions of the protein and they are known to play important roles in mediating protein-protein and protein-ligand interactions [63]. In a number of cases, surface loops in protein sequences due to either enabling (i.e. facilitate interaction) or disabling (prevent interactions) characteristics have been shown to play important role in determining the oligomeric state of proteins [63,64].

Much of the work on PKs thus far has focused on bifidobacteria. The functional enzyme (XFPK) in bifidobacteria is a dimer with its active site located in between the two subunits. Our analyses of the conserved indels found in the bifidobacterial PKs show that at least two of these CSIs (viz. CSI #7 (D461-E462) and CSI #1 (F567-N569)) are located at the subunit interface, and they are indicated to play a role in the formation/stabilization of the protein dimer by means of hydrogen bonding, salt bridge formation or by providing an additional surface for subunit interaction. Studies on the dimerization potentials of the monomeric XFPK proteins, which either contained or lacked these CSIs, show that the docking scores for the XFPK monomers, which contained the CSIs were consistently higher in comparison to those obtained with the corresponding proteins that lacked these two CSIs. These results support the hypothesis that at least some of the CSIs, which distinguish the XFPK homologs of bifidobacteria from the XPK homologs, play an important role in the formation/stabilization of the dimeric form of the XFPK enzyme. In contrast to bifidobacteria, very limited work has been carried out on PKs from other bacteria and no reliable information is available concerning the oligomeric state of the functional XPK enzyme. Because the latter proteins are lacking the CSIs involved in the formation/stabilization of the dimeric protein, it is possible that the PK enzymes found in other microbes may function as monomers, or that the dimers formed in these cases are less stable, thus affecting the ability of the enzyme to bind to different substrates (viz. X5K and F6P). However, besides the CSIs that are indicated to be involved in dimer formation/stabilization, a number of other CSIs differentiating XFPK and XPK homologs are present in other parts/locations in the protein, and their influence on the overall functioning of the XFPK, including its regulation or its ability to recognize both X5K and F6P, remains to be explored. Further work on understanding the functional significance of different identified CSIs on the biochemical activities of the XFPK/XPK homologs should prove very relevant and informative in these regards.

Lastly, our observation that the PKs from bifidobacteria are closely related to those found in the *Coriobacteriales*, and that the PK gene in bifidobacteria was likely acquired from the latter group of microbes by means of HGT shifts our focus to the order *Coriobacteriales*. It is of interest in this regard that similar to the bifidobacteria, members of the order *Coriobacteriales* are also commensal organisms and they constitute significant constituents of the gut microbiota in humans and other animals [32,65,66]. Further similar to the *Bifidobacteriales*, some members of the order *Coriobacteriales* viz. *Atopobium* and *Olsenella* are associated with periodontal/endodontic infections, and the species *Atopobium vaginae* is commonly found (~ 80% of the cases) in bacterial vaginosis [32,67,68]. The order *Coriobacteriales* is a part of the class *Coriobacteriia* [31,32,52]. However, of the two orders that are present in this class, only members from the order *Coriobacteriales* exhibit saccharolytic ability and are able to metabolize glucose and wide variety of other carbohydrates, producing lactate and acetic acid as the main metabolites [31,32]. In contrast, the other order, *Eggerthellales*, is entirely made up of assacharolytic organisms [31] and no PK homolog could be detected in these bacteria. It should also be noted that Tween 80, which is a constituent of the growth medium for bifidobacteria, also exhibits a stimulatory effect on the growth of various *Coriobacteriales* species [3,8,32]. Thus, members of the order *Bifidobacteriales* and *Coriobacteriales* are very similar to each other in terms of their ecological niches, pathogenicity profiles, as well as their ability to utilize different carbohydrates and the metabolite end products produced [3,5,6,8,32,66,69]. In view of these observations and the remarkable similarity in the sequences of PK homologs from these two orders of bacteria, including the shared presence of large numbers of highly specific conserved indels, it is quite likely that the PK homologs from *Coriobacteriales*, similarly to the bifidobacteria, may also be able to recognize and metabolize both X5K and F6P as substrates. Thus, it is possible that *Coriobacteriales* may constitute another group of microbes which are able to metabolize carbohydrates via the “bifid shunt”. Further biochemical investigations in this regard should be of much interest.

## Supporting information

S1 Fig Sequence alignment files of PKs showing other conserved indels that are uniquely shared characteristic of the bifidobacteria and/or *Coriobacteriales* homologs. (PDF)
