# Supplementary material for: Novel molecular, structural and evolutionary characteristics of the phosphoketolases from bifidobacteria and Coriobacteriales
Source: PLoS One. 2017 Feb 17;12(2):e0172176. doi: 10.1371/journal.pone.0172176 (PMC5315409; doi:10.1371/journal.pone.0172176)
Supplement: S1 Fig — (PDF) [file pone.0172176.s001.pdf]

CSI # 4

Group	Species	Accession	744–793
Bifidobacteriales (>50/>50)	Bifidobacterium bifidum	KXS26938	DMVRVNELDRYELTAEALRMIDAD KYADEIQKLEDFRQEAFQFAVDKGYD
	Alloscardovia criceti	WP_018143580	------NI-----------AV--- -F----E---A--T--------N---
	Alloscardovia omnicolens	WP_049217571	------DI-----------AV--- -F-A--E---A--T--------N---
	Bifidobacterium actinocoloniif	WP_033503331	------DM--WC-A-S--ELV--K ---GQ-DEWTK--T--------N---
	Bifidobacterium adolescentis	AAR98784	------NI-----Q---------- ----K-NE--A-----------N---
	Bifidobacterium aesculapii	WP_055427017	-------I---------------- ---AK-DE--K--I--------H---
	Bifidobacterium angulatum	WP_003826697	------NI-----V---------- ----K-NE--A--T--------N---
	Bifidobacterium animalis	WP_004217768	------DM---A-Q-K--EL---- ----K-NE-NE--KT-------N---
	Bifidobacterium asteroides	WP_046323000	------NM--WC-A-S--KLV--G ----Q-D-WTK--D-----------
	Bifidobacterium biavatii	WP_033495618	------NI------------V--E -F--K-NE--A-----------N---
	Bifidobacterium bohemicum	WP_033520339	-------M------------V--- ------A---Q---D----------
	Bifidobacterium bombi	WP_044086681	------DM-----E------V--- ----K-AE--K--KD----------
	Bifidobacterium boum	WP_026502452	------NI------------V-T- ---A--D---A--D-----------
	Bifidobacterium breve	ADF97524	------DM---A-Q-A--KL---- ----K-DE-NA--KK-------N---
	Bifidobacterium callitrichos	WP_043167683	-------I---------------- ----K-NE--A--N--------H---
	Bifidobacterium catenulatum	ADY17517	------NI-----V---------- ----K-NE--A-----------N---
	Bifidobacterium choerinum	WP_024540438	------DM---A-E-Q--EL---- ----K-NE-NE--KK-------N---
	Bifidobacterium commune	SCC79072	------RM--------------EN ------A---Q--ED----------
	Bifidobacterium coryneforme	WP_033498555	------NM--WC-A-S--Q-V--Q ----Q-D-WTK--D-----------
	Bifidobacterium crudilactis	WP_034252558	------NI-----------AV--E -F-SK-DE--K--T----Y-----F-
	Bifidobacterium cuniculi	WP_033516398	------DM---A-Q-----L---- ----K-DE-NA--KK-------N---
	Bifidobacterium gallicum	WP_006294035	------DI-------T-------- ------K------I--Y-----N---
	Bifidobacterium gallinarum	WP_033506218	------DI-----V---------- ---EK-DE--A--T--------N---
	Bifidobacterium indicum	WP_033490190	------NM--WC-A-S--Q-V--Q ----Q-D-WTK--D-----------
	Bifidobacterium kashiwanohense	WP_033501227	------NI-----V---------- ---EK-DE--A-----------N---
	Bifidobacterium longum	AAR98787	------RI---------------- ----K-DE--K--D--------N---
	Bifidobacterium magnum	WP_022859707	------DI-------T-------- --T---D---K--K--YE-T-EN---
	Bifidobacterium merycicum	WP_033521588	------NI-----V---------- ----K-NE--A--T--------N---
	Bifidobacterium minimum	WP_022860755	-------I---------------- ---G--D---K--D-----------
	Bifidobacterium mongoliense	WP_033511011	------DM---A--------V-K- ---AD-D---A--K-----------
	Bifidobacterium moukalabense	WP_034875268	------NI-----V---------- ----K-NE--A--K--------N---
	Bifidobacterium pseudocatenula	KXS25320	------NI-----V---------- ----K-NE--A-----------N---
	Bifidobacterium pseudolongum	WP_026643170	------DM---A-Q-A--K----- ----K-DE-NA---K-------N---
	Bifidobacterium psychraerophil	WP_033496232	------NI-----------AV--E -F-SK-DE--K--TD--------F-
	Bifidobacterium pullorum	WP_033514074	------DI-----V---------- ----K-DE--A--T--------N---
	Bifidobacterium reuteri	WP_044088505	-------I---------------- ---AK-DE--K--D--------N---
	Bifidobacterium ruminantium	WP_026645831	------NI-----Q---------- ----K-NE--A---------------
	Bifidobacterium saeculare	WP_033509310	------DI-----V---------E ----K-DE--A--T---K----N---
	Bifidobacterium saguini	WP_033890392	------RI---------------- ----K-AE--A-----------N---
	Bifidobacterium scardovii	WP_033517588	------NI---------------E ----K-DE--A--K-----------
	Bifidobacterium stellenboschen	WP_034526492	-------I---------------- ------K-----L--------N---
	Bifidobacterium subtile	WP_024462918	------DI------------V--- -F-AD-D---A--D---------F-
	Bifidobacterium thermacidophil	WP_029576306	------NI------------V--- ---A--D---A--D-----------
	Bifidobacterium thermophilum	WP_015450424	------NI------------V--- ---A--D---A--D-----------
	Bifidobacterium tsurumiense	KFJ05925	------DI---------------- ---A--D---A--K-----------
	Gardnerella vaginalis	KXI18594	------DM------------V--A ------K-----L---Y-----L-
	Parascardovia denticolens	WP_006289090	------DM------------V--- ------K-------------------
	Scardovia inopinata	WP_006293010	------DI------------L--- ----D-K-----------Y---N-H-
	Scardovia wiggsiae	WP_007147361	------DI------------L--- ---ED-K-------------------
Coriobacteriales (17/17)	Collinsella aerofaciens	CUP20669	--L-L-NM--WA-A-DV---V--V -F-EQ-DEW-A--T---E--C-E-F-
	Collinsella sp. MS5	WP_040219746	--L-L-NM--WA-A-D----V--Q -W-AQ-EEW-N-----------E---
	Coriobacterium glomerans	WP_013709140	--L-L-RM--WA-A-H---LV-EK RFSTQ-ASW-A--E---R----E---
	Olsenella profusa	WP_021725123	--L-L--M--WA-A-H--ELV-GE R--KR-ADWRA--D--------H---
	Olsenella scatoligenes	WP_059055081	--L-L-HM--WA-A-D---IL--E -W--Q-AEW-Q---D-------N---
	Atopobium parvulum	WP_012809302	--L-L-NM--WA-A-D-------Q -W--Q-DEW-K--T---E---E----
	Atopobium rimae	WP_003149429	--L-L-HM--WA-A-D-------- -WSEQ-VEW-Q--TD--N---E----
	Atopobium vaginae	WP_061103300	----Q-HM--WA-AQD---LV--H -WK-Q-DAW-K--AD----------
Other bacteria (0/>500)	Agreia bicolorata	WP_044440253	--LML-N----H-VIDVIDRVPGLGS-A-ALR-NMQ-A-MR-R-YTREH-E-
	Agrobacterium rhizogenes	WP_034482360	--TVL------H-AL--ISR-PGLAE-VPAAVEG--AKLL-HHAYVRQH-E-
	Agrobacterium sp. 13-626	WP_065707402	--TVL------H-AI--IERVPGLRE-AM-V-NLFR-KLE-HGRYIRQY-E-
	Agrococcus lahaulensis	WP_026373659	---ML-D----R-AMDVVERVPGLAESC--VLEEWA-APAR-RAYTREH-V-
	Bradyrhizobium lablabi	WP_057857553	---VL-Q---FH-AI--IERVPGLGIAA-HVK---R-ALI-HSRYVREH-E-
	Bradyrhizobium liaoningense	WP_061878714	---VL-G----H-AI--IERVPGLAT-A-HVK-QFR-KLI-HSRYVREH-E-
	Cellulomonas carbonis	WP_043608470	---ML-D----R-VIDVIDRVPHLGTRA-ELR-DMV-A-LR-R-YTR-H-E-
	Jiangella gansuensis	WP_051426756	---ML-D----H-VMDVIDRVSGLGSRQ-ALR-RMA-A-LR-R-YTREH-T-
	Microbacterium azadirachtae	WP_045251598	---ML-H----Q-AID-IDRVPGLAQRC-GLR-E-Q-A-LR-RAYTREH-E-
	Rhizobium aegyptiacum	WP_064697053	--TVL------H-AL--IARVPDLAE-VP-V-AD-HAKLDAHRA-VRER-E-
	Rouxiella chamberiensis	WP_045046811	--TVL------H-ALS-IEKVPGLETSA-EAV-LFK-KLE-HYHYVRQY-D-
	Salinibacterium sp. PAMC 21357	WP_010202274	---ML-D----H-VIDV-ERVPGLTSRH-AFW-EMH-T-LH-Q-YTREH-E-
	Shinella sp. GWS1	KOC71484	--AVL-----FH-AI--VERLPVLRE-AEPILAT-REKLV-HKS-VCEH-E-
	Timonella senegalensis	WP_019147697	---ML-D----H-VIDVIDRVPGLGS---GLR--MQ-K-I--RAYTREH-Q-
	Xylanimonas cellulosilytica	WP_012877475	---ML-DM---H-VIDVIDRVPGMASR--GLR-RMQ-A-IR-RAYTREH-D-

Figure A

Partial sequence alignment of PKs showing a 1 aa deletion that is a uniquely shared characteristic of the Bifidobacteria and *Coriobacteriales* homologs. The dashes (-) in all alignments indicate identity with the amino acid shown on the top line.

Group	Species	Accession	224	CSI # 5		CSI # 6	274
**Bifidobacteriales (>50/>50)**	Bifidobacterium bifidum	WP_003812794	ILSRISDEELHEFFHGMGYEPY	E	FVAG	FDD	EDHMSIHRRFAELFESVWDEI
	Alloscardovia criceti	WP_018143580	--A-------------------	-	----	---	--A--------D---T-F---
	Alloscardovia omnicolens	WP_021618496	--A-------------------	-	----	---	--A--------D---T-F---
	Bifidobacterium actinocoloniif	WP_033503331	---------------------H	-	----	---	-S---------D-L---F-D-
	Bifidobacterium adolescentis	WP_046999231	----------------------	-	----	--N	---L-----------T-F---
	Bifidobacterium aesculapii	WP_055427017	----------------------	-	----	---	-------------W--I----
	Bifidobacterium angulatum	WP_045920315	----------------------	-	----	---	----------------IF---
	Bifidobacterium animalis	WP_004217768	--A--------D--R----H--	-	----	--N	---L-----------TIF---
	Bifidobacterium asteroides	WP_015021938	------------Y-K------F	-	----	---	---L-------D-L-T-F-K-
	Bifidobacterium biavatii	WP_033495618	----------------------	-	----	---	---------------N-----
	Bifidobacterium bohemicum	WP_033520339	-----P--------E-------	-	----	---	-------------L-T-F---
	Bifidobacterium bombi	WP_044086681	--A--P-N------E------F	-	----	---	-----------A-L-E-F-K-
	Bifidobacterium boum	WP_026502452	----------------------	-	----	---	-----------D-----F---
	Bifidobacterium breve	ADF97524	--A--------D--R----H--	-	----	--N	---------------TIF---
	Bifidobacterium callitrichos	WP_043167683	----------------------	-	----	---	-----------D-W--I----
	Bifidobacterium catenulatum	WP_003835159	----------------------	-	----	--N	---L-----------T-F---
	Bifidobacterium choerinum	WP_024540438	--A--------D-------H--	-	----	--N	---L-----------TIF---
	Bifidobacterium commune	SCC79072	--A--P--------E-------	-	----	---	--D--------D---E-F-K-
	Bifidobacterium coryneforme	WP_033498555	---------------------F	-	----	---	---I---A-----M-T-F-K-
	Bifidobacterium crudilactis	WP_034252558	----------------------	-	----	---	--SL------SD---T-F---
	Bifidobacterium cuniculi	WP_033516398	--A--------D--R----H--	-	----	--N	---L-----------T-F---
	Bifidobacterium gallicum	WP_006294035	--A-V------D--R-L--H--	-	----	--N	---L-----------TIF---
	Bifidobacterium gallinarum	AAR98786	--A----------------Q--	-	----	---	-----------D---T-F---
	Bifidobacterium indicum	WP_033490190	---------------------F	-	----	---	---I---A-----M-T-F-K-
	Bifidobacterium kashiwanohense	WP_033501227	----------------------	-	----	--N	---L-----------T-F---
	Bifidobacterium longum	AAR98787	----------------------	-	----	---	-------------W-TI----
	Bifidobacterium magnum	WP_022859707	--A-V------D--R-L--H--	-	----	---	---L-----------T-F---
	Bifidobacterium merycicum	WP_033521588	----------------------	-	----	---	----------------IF---
	Bifidobacterium minimum	WP_022860755	-----------D-------D--	-	----	---	---A---H-------T-F---
	Bifidobacterium mongoliense	WP_033511011	-----------D----------	-	----	--N	---L-------D----IF---
	Bifidobacterium moukalabense	WP_034875268	----------------------	-	----	--N	---L-----------T-F---
	Bifidobacterium pseudocatenula	WP_004221672	----------------------	-	----	--N	---L-----------T-F---
	Bifidobacterium pseudolongum	WP_022857642	--A--------D--R----H--	-	----	--N	---------------TIF---
	Bifidobacterium psychraerophil	WP_033496232	----------------------	-	----	---	--SL------SD---T-F---
	Bifidobacterium pullorum	WP_043169083	--A----------------Q--	-	----	---	-----------D---T-F---
	Bifidobacterium reuteri	WP_044088505	----------------------	-	----	---	-------------W-TI----
	Bifidobacterium ruminantium	WP_026645831	----------------------	-	----	--N	---L-----------T-F---
	Bifidobacterium saeculare	WP_033509310	--A----------------Q--	-	----	---	-----------D---T-F---
	Bifidobacterium saguini	WP_033890392	----------------------	-	----	---	-------------W-TI----
	Bifidobacterium scardovii	WP_033517588	----------------------	-	----	---	-S---------D----I----
	Bifidobacterium stellenboschen	WP_034526492	----------------------	-	----	---	----------------I----
	Bifidobacterium subtile	WP_024462918	--A--------D----------	-	----	--N	---L-------D----IF---
	Bifidobacterium thermacidophil	WP_044092029	----------------------	-	----	---	-----------D-----F---
	Bifidobacterium thermophilum	WP_015450424	----------------------	-	----	---	-----------D-----F---
	Bifidobacterium tsurumiense	KFJ05925	----------------------	-	----	---	---L-----------T-F---
	Gardnerella vaginalis	KXI18594	----------------------	-	----	---	---L-------DM--TIF---
	Parascardovia denticolens	WP_006289090	--A--------D--R----N--	-	----	---	-----------D-L-T-F---
	Scardovia inopinata	WP_006293010	--A--------D--R----N--	-	----	---	-----------D-L-T-F---
	Scardovia wiggsiae	WP_007147361	--A--T-----D--S----N--	-	----	---	-----------D-L-T-F---
***Coriobacteriales* (17/17)**	Collinsella aerofaciens	CUP20669	--A-V-----TK--E----K-H	F	-I--	---	-S-A---E---A---Q-F---
	Collinsella stercoris	WP_006719698	--A-------TK--E----K-H	F	----	--N	-S-A---A---AQL-V-FG--
	Coriobacterium glomerans	WP_013709140	--A-------DA--R----A-H	V	----	---	---L-------D-I-Q-FA--
	Olsenella profusa	WP_021725123	--A------RN---R----H--	T	----	---	-PQL-------A-L-W-F---
	Olsenella scatoligenes	WP_059055081	--A----G-RD---R----H--	N	----	--E	---A-------T-L-A-F---
	Atopobium parvulum	WP_012809302	--A----G-RD---R----H--	N	----	---	---A-------A-L-A-FN--
	Atopobium rimae	WP_003149429	-------G-RD---R----H--	T	----	--N	---A-------A---A-F---
	Atopobium vaginae	KMT48306	--A-V-LQ-RDDY-K----R--	T	----	--E	--TL---K--VA---EIFA-L
**Other bacteria (31/>500) (mostly from *Micrococcales*)**	Acidobacterium sp. PMMR2	WP_026441473	--A---T---DAL-R-Y--T--		V---		D-PYTM-QQM-ATL-QCIT--
	Actinokineospora inagensis	WP_026421306	V-A-VP-D--AALMR-Y--K-H		----		S-P-AV-EA--TTVDA-L---
	Actinomadura rifamycini	WP_026402262	V-A--PE---VQLLD-Y--H-L		L---		DEPA-M--KM-AALDQAL---
	Bradyrhizobium elkanii	WP_028164193	--A---H---DQL-R-Y--T--		--E-		DEPEKM-ELM-STLDKIT---
	Calothrix parietina	WP_015195818	V-A---H---ESL-I-Y--K--		--E-		S-PETM-QLM-ATLDT-IE--
	Chlamydia sp. 'Diamant'	WP_032124650	--A---Q---ESL-I-Y--K--		YLE-		S-PE-M--LM--TLDT-IR--
	Chlorobaculum tepidum	WP_010933190	V-A---H---EQLMI-Y--K--		--E-		D-PATM-QMM-ATMDRCF---
	Chlorogloeopsis fritschii	WP_016876026	--A---H---EGL-R-Y--T--		--E-		D-PETM-QDM-ATL-ECVN--
	Clostridium sp. CAG:710	CCZ58578	V-A-VDKK--NDY-K-L-WH--		Y-E-		D-PIQM-EIM--VL-L-IK--
	Cystobacter fuscus	WP_002632470	L-A--DPD--EAL-R-Y--H--		VLE-		D-PRTM-Q-M--VLDTLYR--
	Fischerella muscicola	WP_016868244	--A---H---EDLYK-Y--T--		--E-		S-PE-M-QAM-ATLDHCIS--
	Mesorhizobium loti	WP_051429804	V-A---P---EAL-R-Y--D-I		--E-		-EPEIM-QKM-AAM-GAFQR-
	Neorhizobium galegae	WP_046664687	L-A-VG--D-DHL-R-Y----F		--E-		HEPEKM--QM-AVLDG-F-R-
	Nocardia pneumoniae	WP_051022619	V-A--D-D--DHLLR-F-HT--		L---		S-PEQM-QA--AALDRCL---
	Roseburia faecis	WP_022045178	--A---H---EH--D-C-WK--		--E-		D-P-TM--KM--TLDT-M---
	Roseburia intestinalis	WP_015522202	--A---R---EH--D-C-WK--		--E-		DEP-DM-SKM-AALDQAM---

Figure B

Partial sequence alignment of PKs showing two conserved indels (highlighted) that are primarily found in the PK homologs from from Bifidobacteria and *Coriobacteriales*.

Group	Organism	Accession	496	CSI # 3	538
***Bifidobacteriales* (>50/>50)**	Bifidobacterium bifidum	WP_003812794	GIWSSYESFVHVIDSMLNQHAKWLEAT	V	REIPWRKPIASMNLL
	Alloscardovia criceti	WP_018143580	---------------------------	-	---------S-----
	Alloscardovia omnicolens	WP_049217571	---------------------------	-	---------S-----
	Bifidobacterium adolescentis	AAR98784	---------------------------	-	---------S-----
	Bifidobacterium aesculapii	WP_055427017	---------------------------	-	---------------
	Bifidobacterium angulatum	WP_003826697	---------------------------	-	---------S-----
	Bifidobacterium animalis	WP_052826255	-M--T----A-----------------	-	---------S-V---
	Bifidobacterium asteroides	WP_046323000	----------------I----------	-	----------GL---
	Bifidobacterium biavatii	WP_033495618	---------------------------	-	---------S-----
	Bifidobacterium bohemicum	WP_033520339	---------------------------	-	-------------M-
	Bifidobacterium bombi	WP_044086681	----T----A-----------------	-	-----------I-M-
	Bifidobacterium boum	WP_026502452	---------------------------	-	---------S-----
	Bifidobacterium breve	ADF97524	---------------------------	-	---------S-V---
	Bifidobacterium callitrichos	WP_043167683	---------------------------	-	---------------
	Bifidobacterium catenulatum	ADY17517	---------------------------	-	---------S-----
	Bifidobacterium choerinum	WP_024540438	---------------------------	-	---------S-V---
	Bifidobacterium commune	SCC79072	------------V--------------	-	-----------L-M-
	Bifidobacterium coryneforme	WP_033498555	------------V---I----------	-	----------G----
	Bifidobacterium crudilactis	WP_034252558	---------------------------	-	---------S-V---
	Bifidobacterium cuniculi	WP_033516398	---------------------------	-	---------S-----
	Bifidobacterium gallicum	WP_006294035	---------------------------	-	---------S-----
	Bifidobacterium gallinarum	WP_033506218	---------------------------	-	---------S-----
	Bifidobacterium indicum	WP_033490190	------------V---I----------	-	----------G----
	Bifidobacterium kashiwanohense	WP_039197875	---------------------------	-	---------S-----
	Bifidobacterium longum	WP_060621289	---------------------------	-	---------------
	Bifidobacterium magnum	WP_034250240	---------------------------	-	---------S-----
	Bifidobacterium merycicum	WP_033521588	---------------------------	-	---------S-----
	Bifidobacterium mongoliense	WP_033511011	-V-------------------------	-	---------S-V---
	Bifidobacterium pseudocatenula	WP_034880609	---------------------------	-	---------S-----
	Bifidobacterium pseudolongum	WP_022857642	-M--T----A-----------------	-	---------S-V---
	Bifidobacterium psychraerophil	WP_033496232	---------------------------	-	---------S-V---
	Bifidobacterium pullorum	WP_033514074	---------------------------	-	---------S-----
	Bifidobacterium reuteri	WP_044088505	---------------------------	-	---------------
	Bifidobacterium ruminantium	WP_026645831	---------------------------	-	---------S-----
	Bifidobacterium saguini	WP_033890392	---------------------------	-	---------------
	Bifidobacterium scardovii	WP_033517588	---------------------------	-	---------S-----
	Bifidobacterium stellenboschen	WP_034526492	---------------------------	-	---------------
	Bifidobacterium subtile	WP_024462918	--------------------------V	-	---------S-----
	Bifidobacterium thermacidophil	WP_029576306	---------------------------	-	---------S-----
	Bifidobacterium thermophilum	WP_044279946	---------------------------	-	---------S-----
	Bifidobacterium tsurumiense	WP_026641992	---------------------------	-	---------S-----
	Gardnerella vaginalis	KXI18594	---------------------------	-	---------S-V---
	Parascardovia denticolens	WP_006291758	---------------------------	-	---------S-V---
	Scardovia inopinata	WP_006293010	---------------------------	-	---------S-V---
	Scardovia wiggsiae	WP_007147361	---------------------------	-	---------S-V---
***Coriobacteriales* (17/17)**	Collinsella aerofaciens	CUP20669	-V----------V---V---C------	K	------A--SGL-I-
	Collinsella stercoris	WP_006719698	-V----------V---V---C------	K	------A--SGL-I-
	Olsenella profusa	WP_021725123	-V-------I------V----------	-	-H----A---GL-I-
	Olsenella scatoligenes	WP_059055081	-L---------IV---V---C------	-	---------SGL-M-
	Coriobacterium glomerans	WP_013709140	------------V---I---C------	-	---D-----SGL-I-
	Atopobium parvulum	WP_035432135	-----------IV---I----------	-	-H-------SAL--V
	Atopobium rimae	WP_003149429	-----------IV---I----------	-	-H-------SAV-V-
	Atopobium vaginae	WP_061103300	------------V---I---C-----C	-	-H----R--SGI---
**Other bacteria (24/>500)**	Actinotalea ferrariae	WP_052022328	-LF-C--A-I-IV---F-------KV-		------R----L-Y-
	Allokutzneria albata	WP_030433355	-LF-C--A-I-IV-A---------KV-		-T----R--S-L-Y-
	Aureimonas frigidaquae	WP_062228351	-LF-C--A-I-IV---F-------KVS		--L---A--S-L-Y-
	Demequina aestuarii	WP_062131690	-LFT---A-I-IV---F-------KTS		--L---R--G-L-Y-
	Glycomyces arizonensis	WP_026925238	-LFT---A-I-IV---F--------S-		D-----R----L-Y-
	Herbidospora yilanensis	WP_062356451	-LFNC--A-I-IV-A-F-------DTA		------R-V--L-Y-
	Martelella mediterranea	WP_018066959	-LF-C--A-I-I----F-------KT-		-TM------S-L-Y-
	Microbacterium azadirachtae	WP_045251598	-LFTC--A-I-IV---F-------KV-		-R----R--S-L-Y-
	Nevskia soli	WP_029920026	-LF-C--A-I-I------------KV-		-D---------L-Y-
	Pantholops hodgsonii	XP_005979086	-FF-C--A-I-I----F-------KV-		--LE-----S-L-Y-
	Pedosphaera parvula	WP_007417581	-FF-C--A-I-IV---F----------		-H------V--L-Y-
	Pseudonocardia autotrophica	WP_037045879	-LFT---A-I-IV-----------KT-		-A----A----L-Y-
	Silvibacterium bohemicum	WP_050059569	-FF-C--A-I-I----F-------KV-		-DL--------L-Y-
	Solirubrobacter soli	WP_028064166	-VFN---A-I-IV---F-------KS-		-H----R-V--L-Y-
	Sulfurifustis variabilis	BAU49719	-LF-C--A-I-IV---F-------KV-		------R----L-Y-
	Synechocystis sp. PCC 7509	WP_009633636	-FF-C--A-I-I------------KT-		-H----R----L-Y-
	Verrucosispora maris	WP_013732407	--FT---A-I-IV---V-------KV-		-D----L-V--L-Y-
	Zavarzinella formosa	WP_020468825	-FF-C--A---IV---V-------KVC		--V---R-V--L-Y-

Figure C

Partial sequence alignment of PKs showing a conserved indel that is mainly limited to the PK homologs from from Bifidobacteria and *Coriobacteriales*.

CSI # 12

Group	Organism	Accession	37	CSI # 12 (highlighted)	80
**_Collinsella, Coriobacterium and Olsenella_ (10/11)**	Collinsella aerofaciens	CUP20669	GQIYLRSNPLMRADF	VDDKTGEARDF	GRPDVKHRLVGHWGTTPG
	Collinsella sp. CAG:289	CDD84211	------------K--	--E----V---	------------------
	Collinsella sp. GD3	WP_026089019	------------K--	--E----V---	--A---------------
	Collinsella sp. MS5	WP_040219746	------------K--	--E----V---	------------------
	Collinsella stercoris	WP_006719698	------------E-W	T-A---KK---	--V---------------
	Coriobacterium glomerans	WP_013709140	------------S--	--E----T---	--E---------------
	Olsenella profusa	WP_021725123	------------D--	T-EG--QV-P-	--E---------------
	Olsenella scatoligenes	WP_059055081	---------------	KNQDGPD G-	T-E---------------
	Olsenella sp. DNF00959	WP_062531778	------------D--	--E-S-QP---	--A---------------
	Olsenella sp. oral taxon 809	WP_009278622	------------D--	--E-S-QP---	--A---------------
**_Atopobium_ (0/5)**	Olsenella sp. SIT9	WP_058270697	------D-----G--		S-E---------------
	Atopobium parvulum	WP_035432135	-----------KDG-		S-E---------------
	Atopobium rimae	WP_003149429	-----------KEG-		N-E---------------
	Atopobium sp. BS2	WP_035434992	-----------KDG-		S-E---------------
	Atopobium sp. ICM42b	WP_035427052	-----------KDG-		S-E---------------
	Atopobium vaginae	WP_006302307	-----------KG--		N-D-I-Y-----------
**_Bifidobacteriales_ (0/>50)**	Alloscardovia criceti	WP_018143580	-----------KEP-		T-E---------------
	Alloscardovia omnicolens	WP_021618496	-----------KEP-		T-E---------------
	Bifidobacterium actinocoloniif	WP_033503331	------------EP-		T-E---------------
	Bifidobacterium adolescentis	WP_046999231	-----------KEP-		T-E---------------
	Bifidobacterium aesculapii	WP_055427017	-----------KEP-		T-E---------------
	Bifidobacterium angulatum	WP_003826697	-----------K-P-		T-E---------------
	Bifidobacterium animalis	WP_004217768	-----------KEP-		T-D---------------
	Bifidobacterium asteroides	WP_015021938	-----------KEP-		T-E---------------
	Bifidobacterium bifidum	WP_003812794	-----------KEP-		T-E---------------
	Bifidobacterium bombi	WP_044086681	-----------KEP-		T-E---------------
	Bifidobacterium boum	WP_026502452	-----------KEP-		T-E---------------
	Bifidobacterium breve	ADF97524	-----------KEP-		T-D---------------
	Bifidobacterium callitrichos	WP_043167683	-----------KEP-		T-E---------------
	Bifidobacterium commune	*SCC79072*	*------D----KEP-*		*T-K---Y-----------*
	Bifidobacterium coryneforme	*WP_033498555*	*-----------KEP-*		*T-E---------------*
	Bifidobacterium crudilactis	WP_034252558	-----------KEP-		T-E---------------
	Bifidobacterium cuniculi	WP_033516398	-----------KEP-		T-D---------------
	Bifidobacterium gallicum	WP_006294035	-----------KEP-		T-D---Y-----------
	Bifidobacterium gallinarum	AAR98786	-----------KEP-		T-N---------------
	Bifidobacterium indicum	WP_033490190	-----------KEP-		T-E---------------
	Bifidobacterium kashiwanohense	WP_033501227	-----------KEP-		T-E---------------
	Bifidobacterium longum	AAR98787	-----------KEP-		T-E---------------
	Bifidobacterium magnum	WP_022859707	-----------KEP-		T-D---Y-----------
	Bifidobacterium merycicum	WP_033521588	-----------K-P-		T-E---------------
	Bifidobacterium pseudocatenula	WP_004221672	-----------KEP-		T-E---------------
	Bifidobacterium pseudolongum	WP_034883174	-----------KEP-		T-D---------------
	Bifidobacterium psychraerophil	WP_033496232	-----------KEP-		T-E---------------
	Bifidobacterium pullorum	WP_033514074	-----------KEP-		T-N---------------
	Bifidobacterium ruminantium	WP_026645831	-----------KEP-		T-E---------------
	Bifidobacterium saeculare	WP_033509310	-----------KEP-		T-N---------------
	Bifidobacterium saguini	WP_033890392	-----------KEP-		T-E---------------
	Bifidobacterium scardovii	WP_033517588	-----------KEP-		T-E---------------
	Bifidobacterium stellenboschen	WP_034526492	-----------KEP-		T-E---------------
	Bifidobacterium subtile	WP_024462918	-----------KEP-		T-E---------------
	Bifidobacterium thermacidophil	WP_029576306	-----------KEP-		T-E---------------
	Bifidobacterium thermophilum	WP_015450424	-----------KEP-		T-E---------------
	Bifidobacterium tsurumiense	KFJ05925	-----------KEP-		T-E---------------
	Scardovia inopinata	WP_006293010	-----------KEP-		T-E---------------
	Scardovia wiggsiae	WP_007147361	-----------KEP-		T-E---------------
	Parascardovia denticolens	WP_006289090	-----------KEP-		T-E---------------
**Other bacteria (0/>250)**	Cellulomonas gilvus	WP_013882940	-----LD---L-EPL		T-DH--P--L--------
	Beutenbergia cavernae	WP_015883531	-----LA---L-TPL		T-D---P--L--------
	Dermacoccus nishinomiyaensis	WP_038569420	-----LD---L-EPL		T-DN-----L--------
	Mycobacterium iranicum	WP_064283518	-----LN---L-TPL		T-E---P--L--------
	Atopococcus tabaci	WP_028274318	-----KD---LKRPL		EVE---ASPI-----V--
	Bacillus acidicola	WP_066265096	--L--KD--ML-NPL		KAA---IKPI-----IA-
	Blautia obeum	CUQ04075	--L--LD---L-EPL		TMDQI-KKI------V--
	Clostridium acetobutylicum	WP_034583456	--L--LD---L-EPL		KPEHL-RKV------I--
	Coprococcus comes	CUN08435	--L--LD---LKKPL		TMDQI-KKI------V--
	Dorea longicatena	CUN89296	--L--LD---LKEPL		TMDQI-KKI------V--
	Enterococcus faecalis	WP_016619665	-----KD---L-RPI		EEK-L-VNPI-----IA-
	Leuconostoc mesenteroides	WP_002815143	--L--LD---LKEKL		TAEQ--IHPI-----I-S
	Melissococcus plutonius	WP_048589794	--L--KD---LKRKI		RSE---YHPI-----IA-
	Roseburia intestinalis	WP_015522202	A-L--LD---L-EPL		TMDHI-KKI------V--
	Streptococcus sobrinus	WP_019787722	A-M--KD---L-RPV		TKE---VHPI-----IA-
	Tetragenococcus halophilus	WP_014123666	A--F--D---L-RPL		EKE-I-INPI-----IS-

Figure D

Partial sequence alignment of PKs showing a conserved indel (highlighted) that is uniquely found in homologs from the genera *Collinsella, Olsenella* and *Coriobacterium* spp.
